# Supplementary material for: The Prevalence of Mild, Moderate, and Severe Nomophobia Symptoms: A Systematic Review, Meta-Analysis, and Meta-Regression
Source: Behav Sci (Basel). 2022 Dec 30;13(1):35. doi: 10.3390/bs13010035 (PMC9854858; doi:10.3390/bs13010035)
Supplement: Supplementary file 1 [file behavsci-13-00035-s001.zip › List of Supplumental Files.pdf]

List of Supplumental Files

1. **Supplemental S1**: Traffic light plot of the risk of bias assessment
2. **Supplemental S2**: Forest plot of the prevalence of all nomophobia symptoms
3. **Supplemental S3**: Funnel plot of the prevalence of all nomophobia symptoms
4. **Supplemental S4**: Galbraith plot of the prevalence of all nomophobia symptoms
5. **Supplemental S5**: DOI plot of the prevalence of all nomophobia symptoms
6. **Supplemental S6**: Subgroup forest plot of the prevalence of all nomophobia symptoms by country
7. **Supplemental S7**: Subgroup forest plot of the prevalence of all nomophobia symptoms by culture
8. **Supplemental S8**: Subgroup forest plot of the prevalence of all nomophobia symptoms by population
9. **Supplemental S9**: Subgroup forest plot of the prevalence of all nomophobia symptoms by tool
10. **Supplemental S10**: Subgroup forest plot of the prevalence of all nomophobia symptoms by year of data collection
11. **Supplemental S11**: Forest plot of the prevalence of mild nomophobia symptoms
12. **Supplemental S12**: Subgroup forest plot of the prevalence of mild nomophobia symptoms by country
13. **Supplemental S13**: Subgroup forest plot of the prevalence of mild nomophobia symptoms by culture
14. **Supplemental S14**: Subgroup forest plot of the prevalence of mild nomophobia symptoms by population
15. **Supplemental S15**: Subgroup forest plot of the prevalence of mild nomophobia symptoms by tool
16. **Supplemental S16**: Subgroup forest plot of the prevalence of mild nomophobia symptoms by year of data collection
17. **Supplemental S17**: Forest plot of the prevalence of moderate nomophobia symptoms
18. **Supplemental S18**: Subgroup forest plot of the prevalence of moderate nomophobia symptoms by country
19. **Supplemental S19**: Subgroup forest plot of the prevalence of moderate nomophobia symptoms by culture
20. **Supplemental S20**: Subgroup forest plot of the prevalence of moderate nomophobia symptoms by population
21. **Supplemental S21**: Subgroup forest plot of the prevalence of moderate nomophobia symptoms by tool
22. **Supplemental S22**: Subgroup forest plot of the prevalence of moderate nomophobia symptoms by year of data collection
23. **Supplemental S23**: Forest plot of the prevalence of severe nomophobia symptoms
24. **Supplemental S24**: Subgroup forest plot of the prevalence of severe nomophobia symptoms by country
25. **Supplemental S25**: Subgroup forest plot of the prevalence of severe nomophobia symptoms by culture
26. **Supplemental S26**: Subgroup forest plot of the prevalence of severe nomophobia symptoms by population
27. **Supplemental S27**: Subgroup forest plot of the prevalence of severe nomophobia symptoms by tool
28. **Supplemental S28**: Subgroup forest plot of the prevalence of severe nomophobia symptoms by year of data collection
