# Supplementary material for: The Prevalence of Mild, Moderate, and Severe Nomophobia Symptoms: A Systematic Review, Meta-Analysis, and Meta-Regression
Source: Behav Sci (Basel). 2022 Dec 30;13(1):35. doi: 10.3390/bs13010035 (PMC9854858; doi:10.3390/bs13010035)
Supplement: Supplementary file 1 [file behavsci-13-00035-s001.zip › Supp S1.pdf]

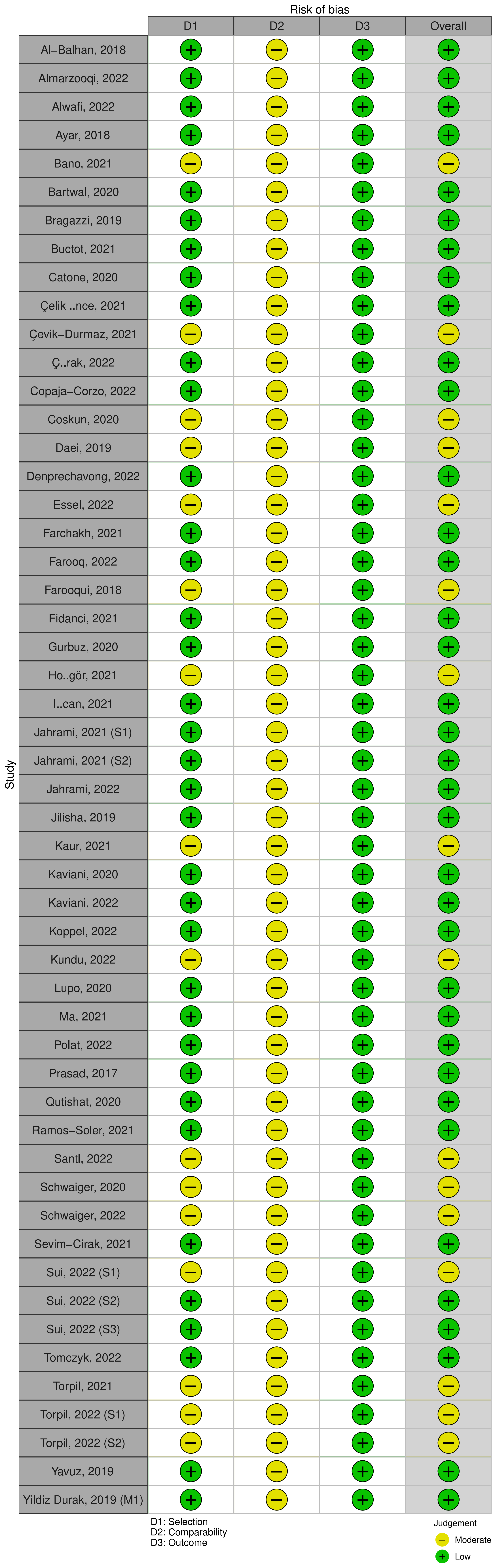
Risk of bias
D1
D2
D3
Overall
Study
Al–Balhan, 2018
Almarzooqi, 2022
Alwafi, 2022
Ayar, 2018
Bano, 2021
Bartwal, 2020
Bragazzi, 2019
Buctot, 2021
Catone, 2020
Çelik ..nce, 2021
Çevik–Durmaz, 2021
Ç..rak, 2022
Copaja–Corzo, 2022
Coskun, 2020
Daei, 2019
Denprechavong, 2022
Essel, 2022
Farchakh, 2021
Farooq, 2022
Farooqui, 2018
Fidanci, 2021
Gurbuz, 2020
Ho..gör, 2021
I..can, 2021
Jahrami, 2021 (S1)
Jahrami, 2021 (S2)
Jahrami, 2022
Jilisha, 2019
Kaur, 2021
Kaviani, 2020
Kaviani, 2022
Koppel, 2022
Kundu, 2022
Lupo, 2020
Ma, 2021
Polat, 2022
Prasad, 2017
Qutishat, 2020
Ramos–Soler, 2021
Santl, 2022
Schwaiger, 2020
Schwaiger, 2022
Sevim–Cirak, 2021
Sui, 2022 (S1)
Sui, 2022 (S2)
Sui, 2022 (S3)
Tomczyk, 2022
Torpil, 2021
Torpil, 2022 (S1)
Torpil, 2022 (S2)
Yavuz, 2019
Yildiz Durak, 2019 (M1)
D1: Selection
D2: Comparability
D3: Outcome
Judgement
Moderate
Low
