# Supplementary figures and images for: The Prevalence of Mild, Moderate, and Severe Nomophobia Symptoms: A Systematic Review, Meta-Analysis, and Meta-Regression
Source: Behav Sci (Basel). 2022 Dec 30;13(1):35. doi: 10.3390/bs13010035 (PMC9854858; doi:10.3390/bs13010035)

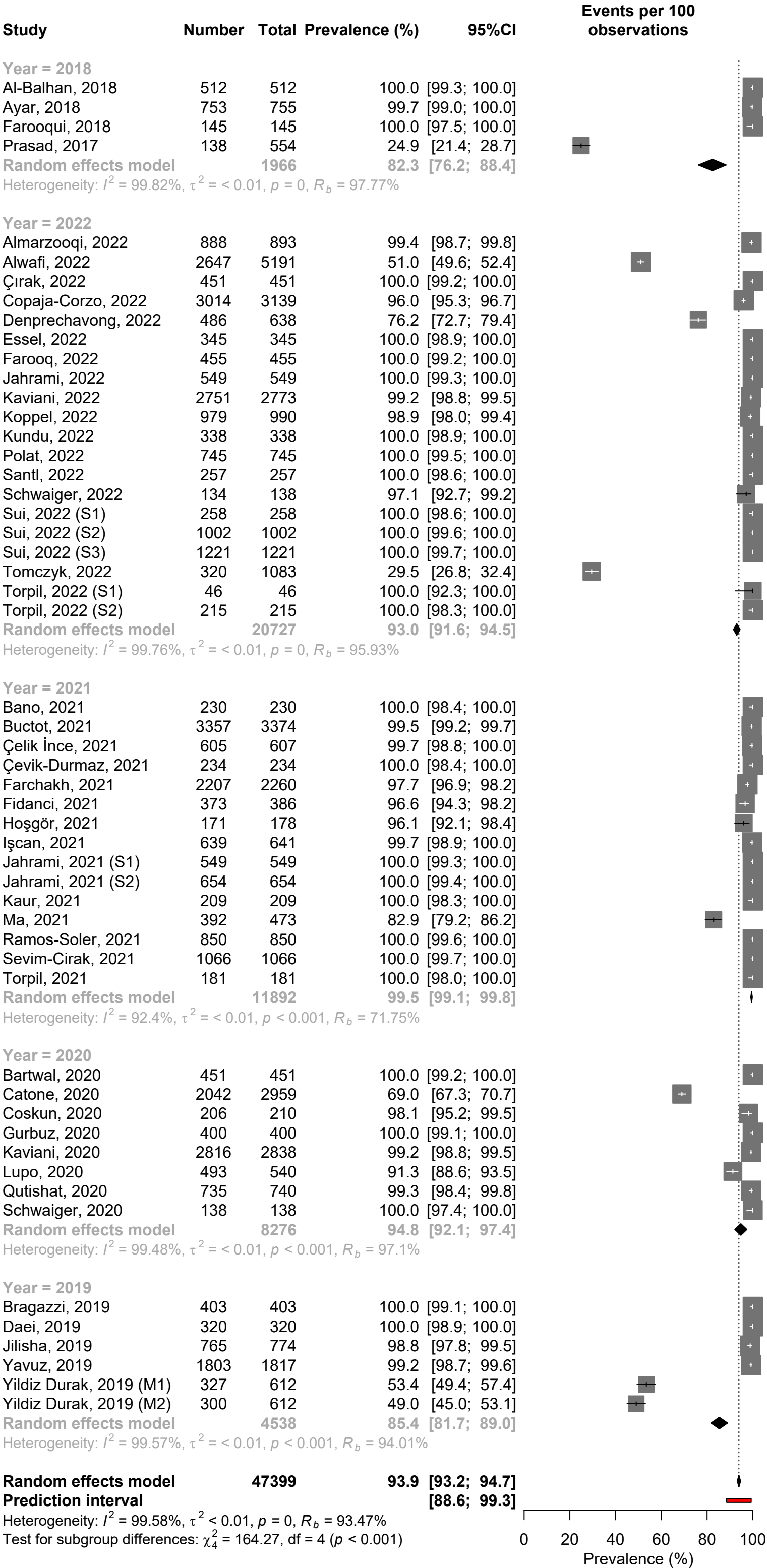

Supplement: Supplementary file 1 [file behavsci-13-00035-s001.zip › Supp S10.pdf]

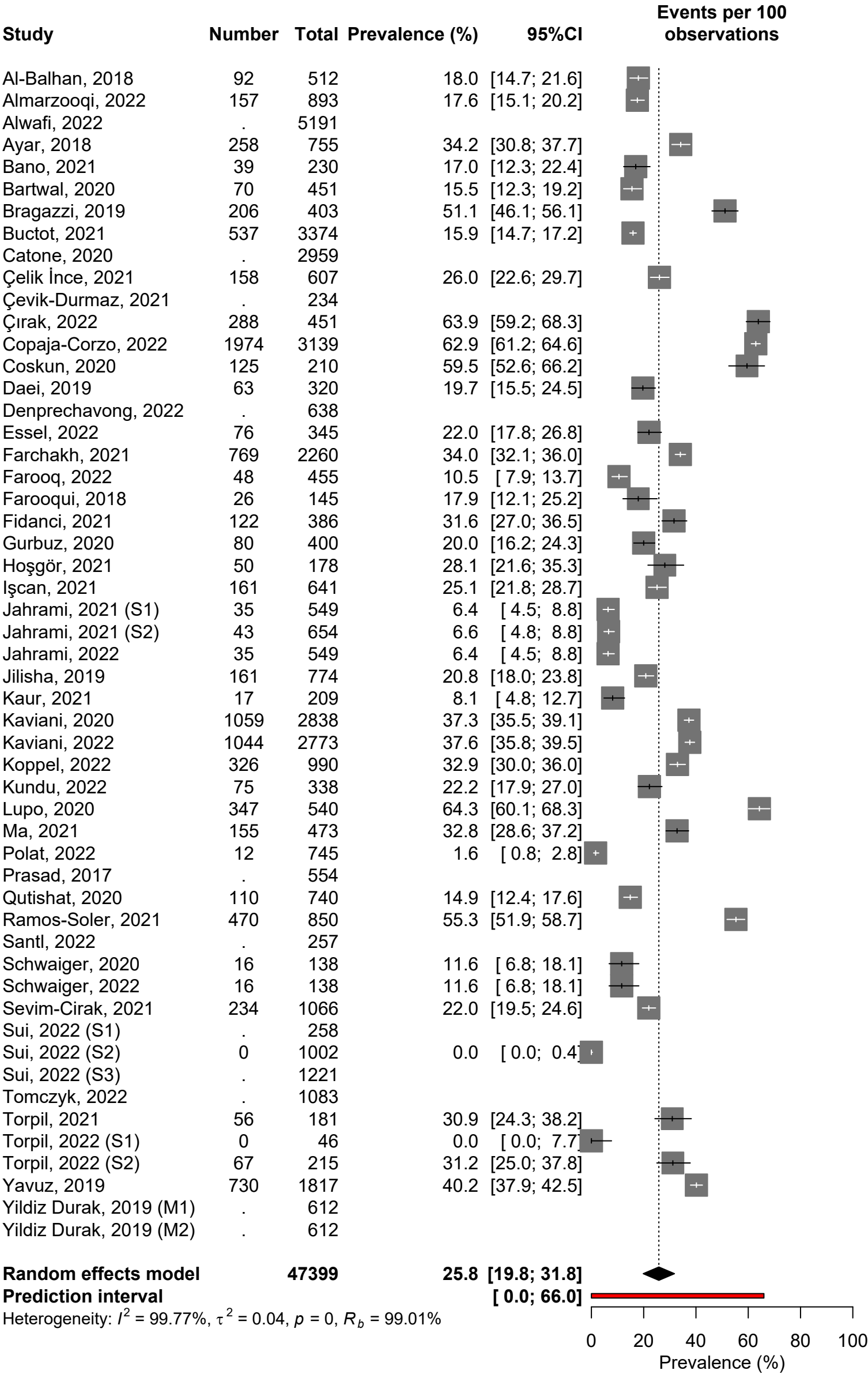

Supplement: Supplementary file 1 [file behavsci-13-00035-s001.zip › Supp S11.pdf]

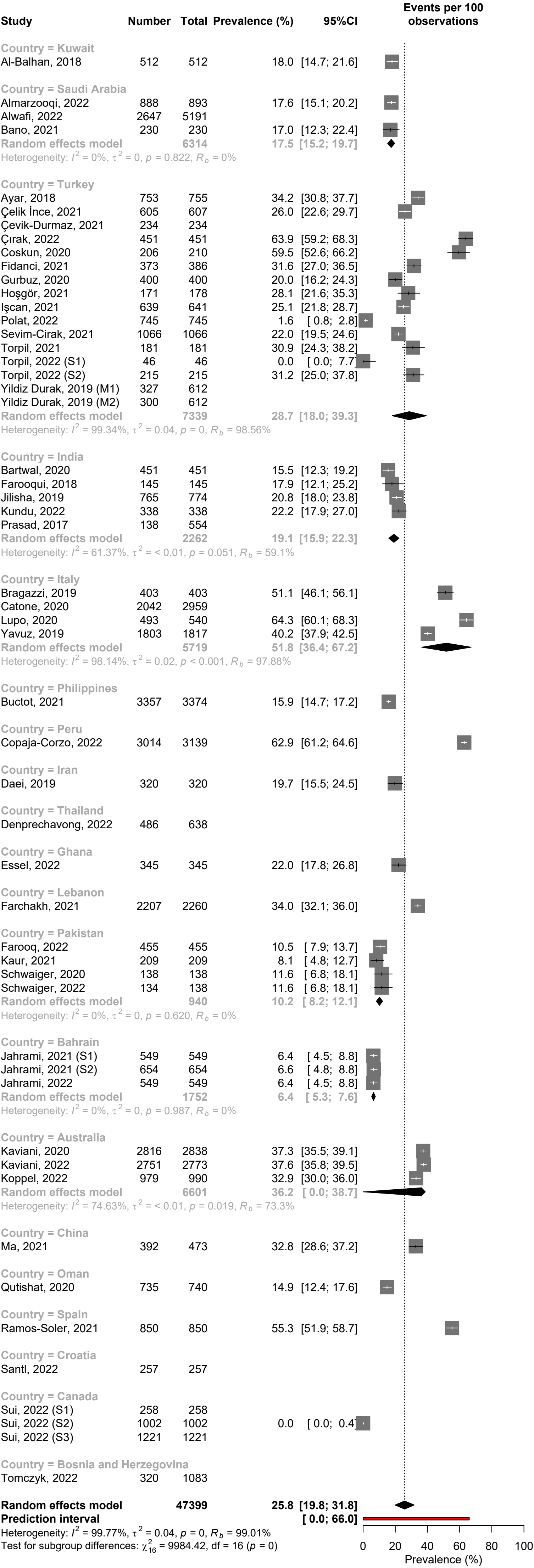

Supplement: Supplementary file 1 [file behavsci-13-00035-s001.zip › Supp S12.pdf]

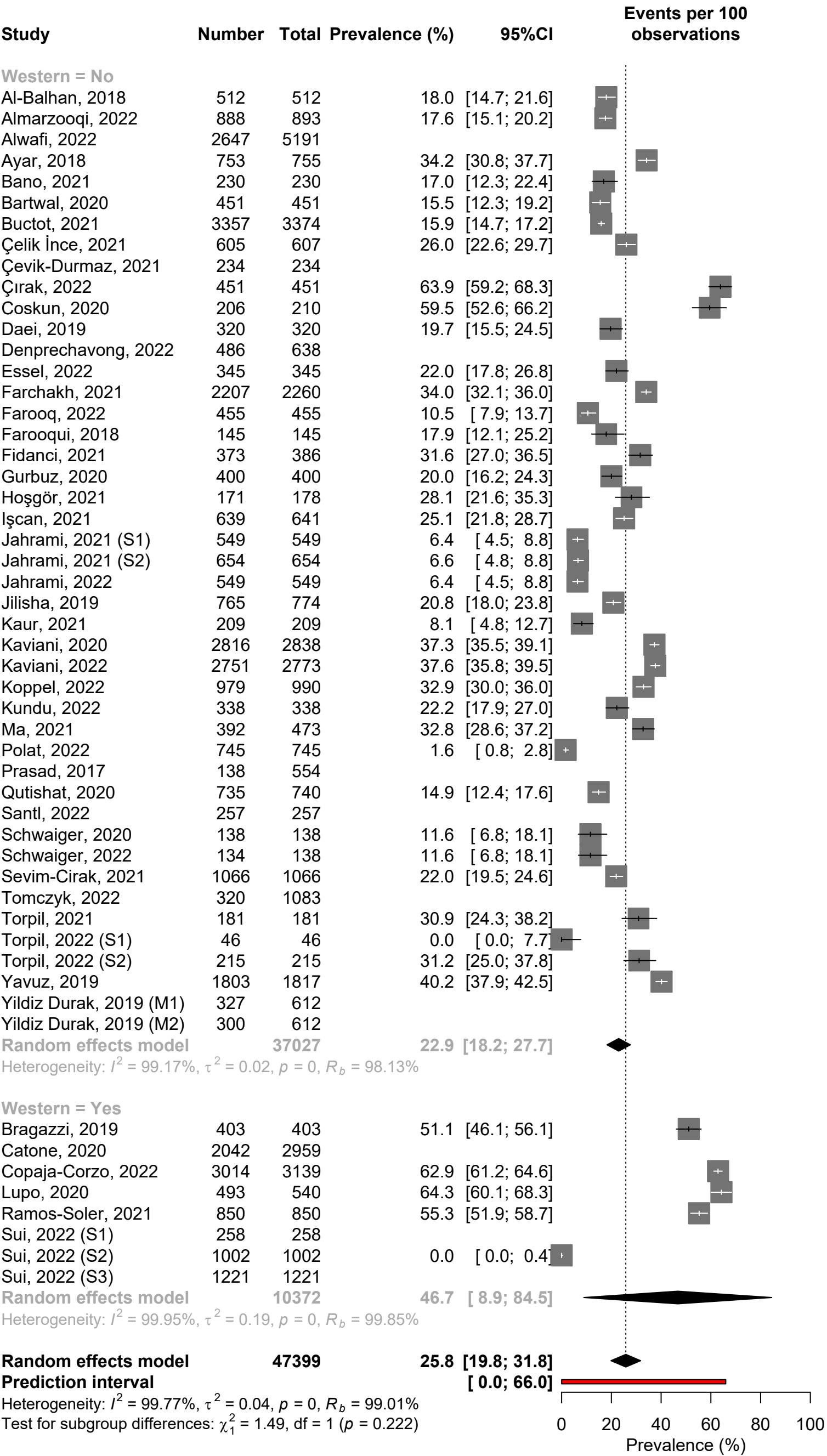

Supplement: Supplementary file 1 [file behavsci-13-00035-s001.zip › Supp S13.pdf]

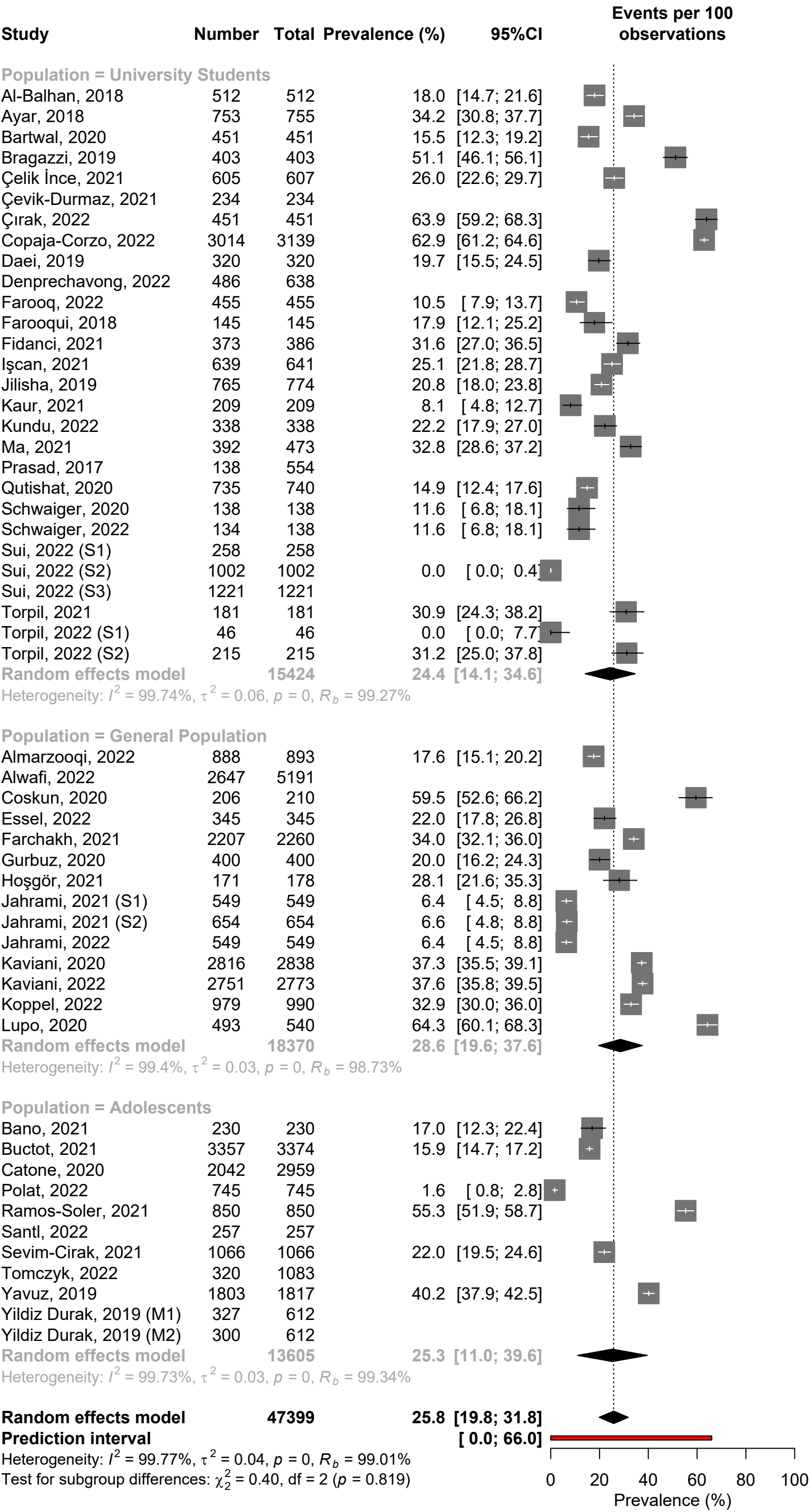

Supplement: Supplementary file 1 [file behavsci-13-00035-s001.zip › Supp S14.pdf]

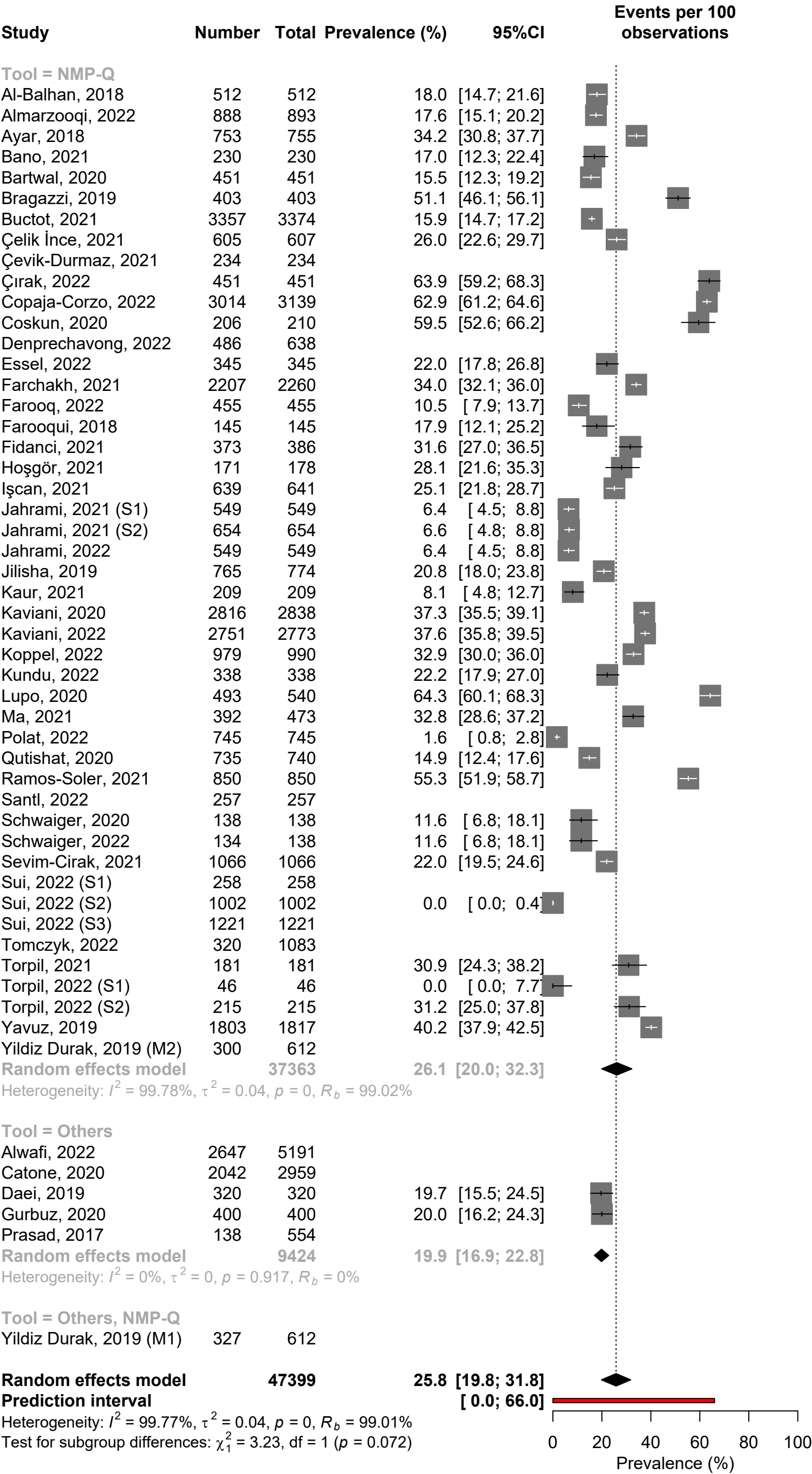

Supplement: Supplementary file 1 [file behavsci-13-00035-s001.zip › Supp S15.pdf]

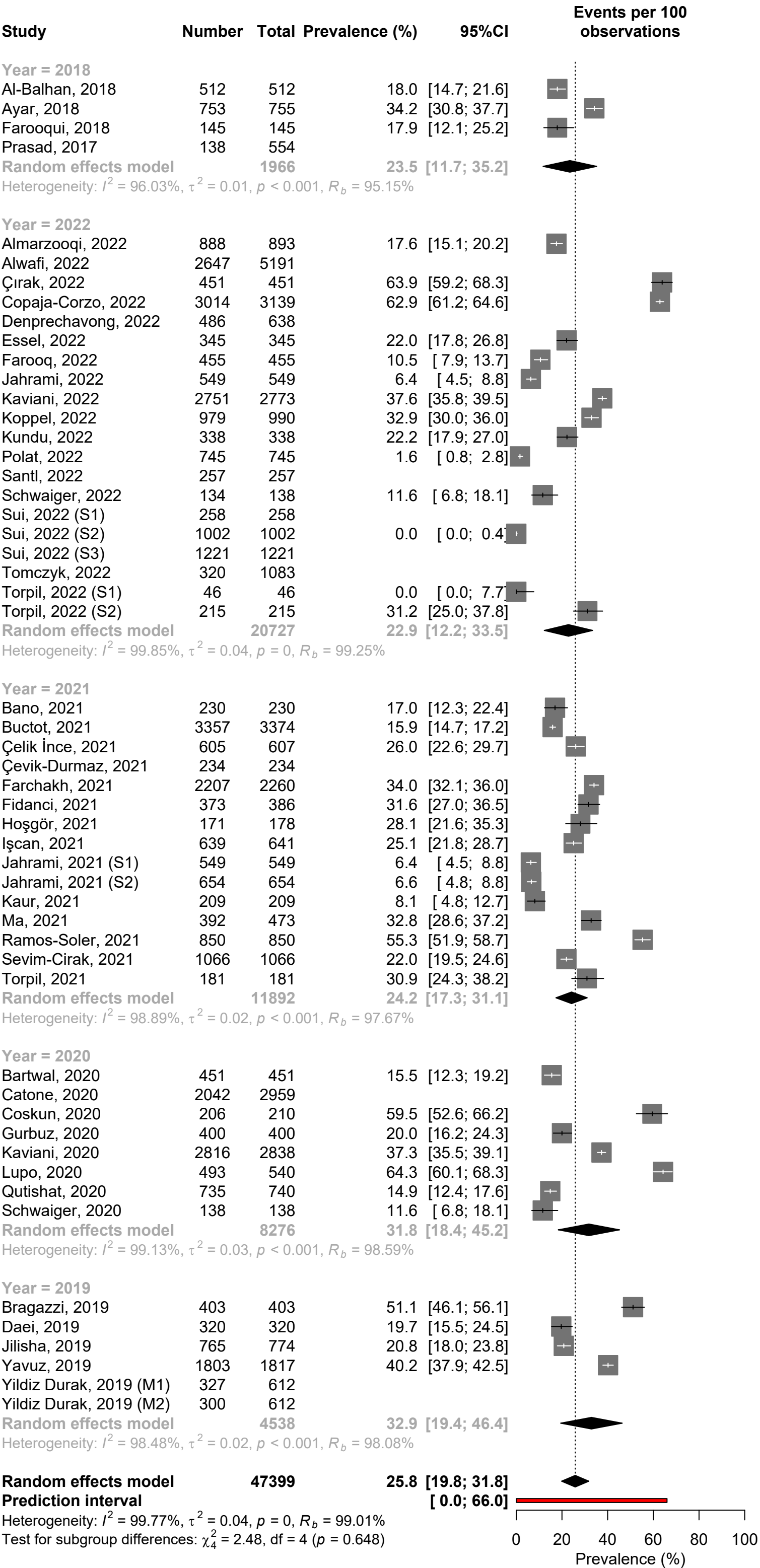

Supplement: Supplementary file 1 [file behavsci-13-00035-s001.zip › Supp S16.pdf]

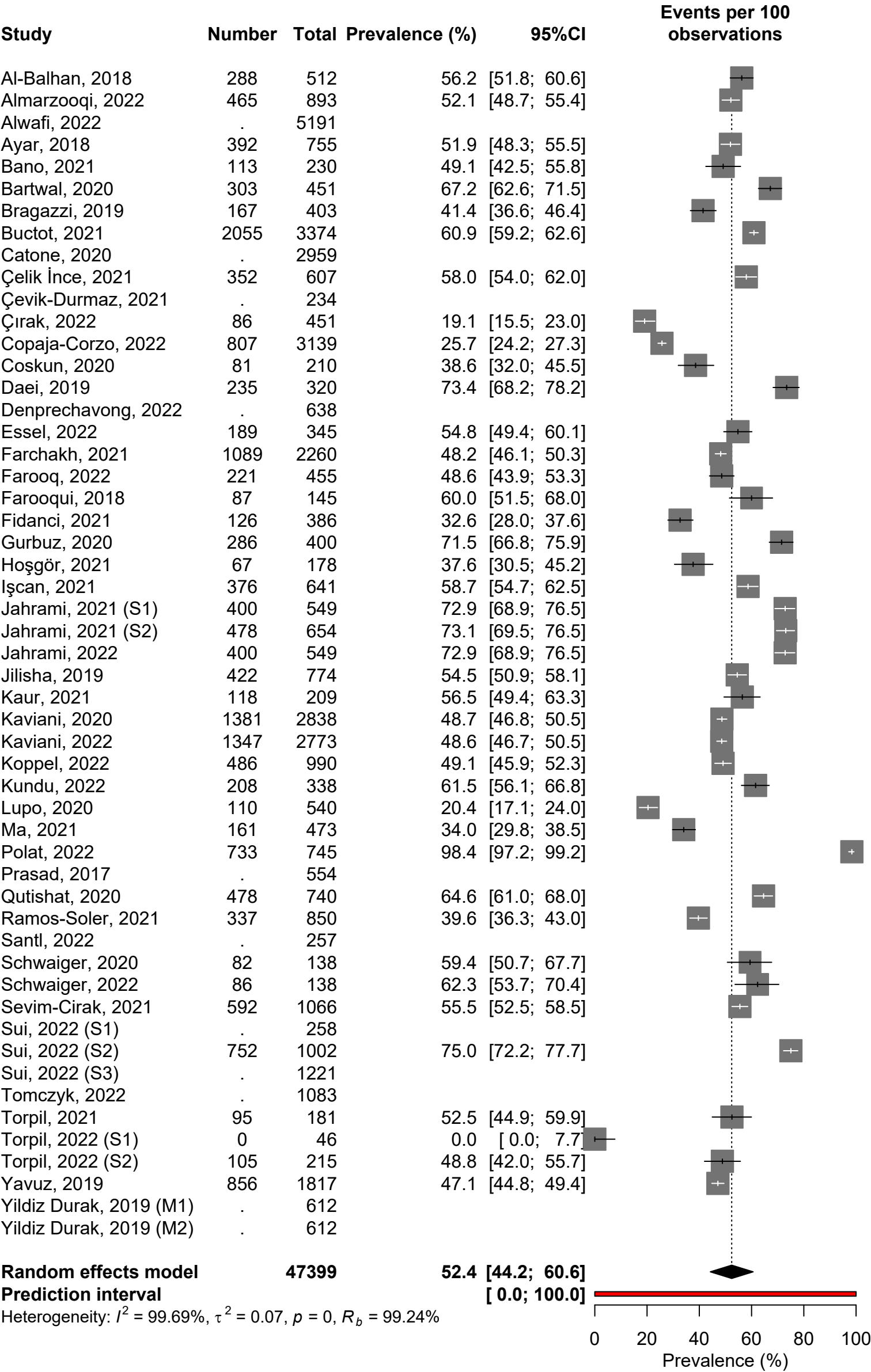

Supplement: Supplementary file 1 [file behavsci-13-00035-s001.zip › Supp S17.pdf]

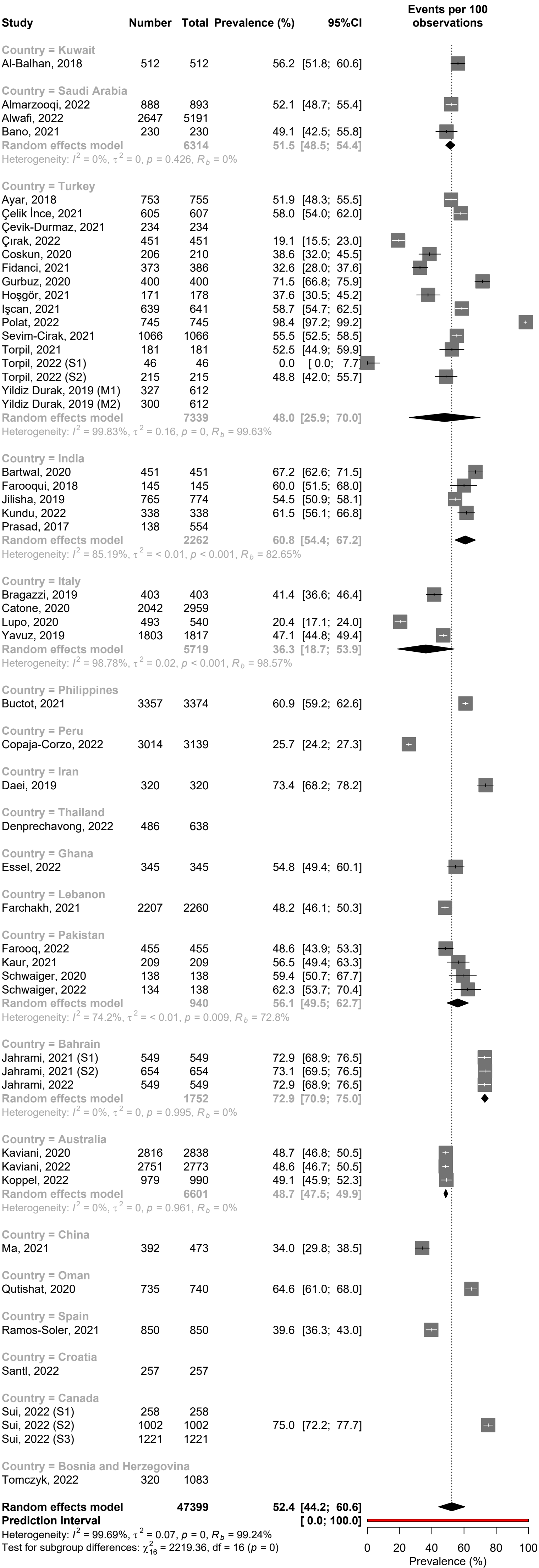

Supplement: Supplementary file 1 [file behavsci-13-00035-s001.zip › Supp S18.pdf]

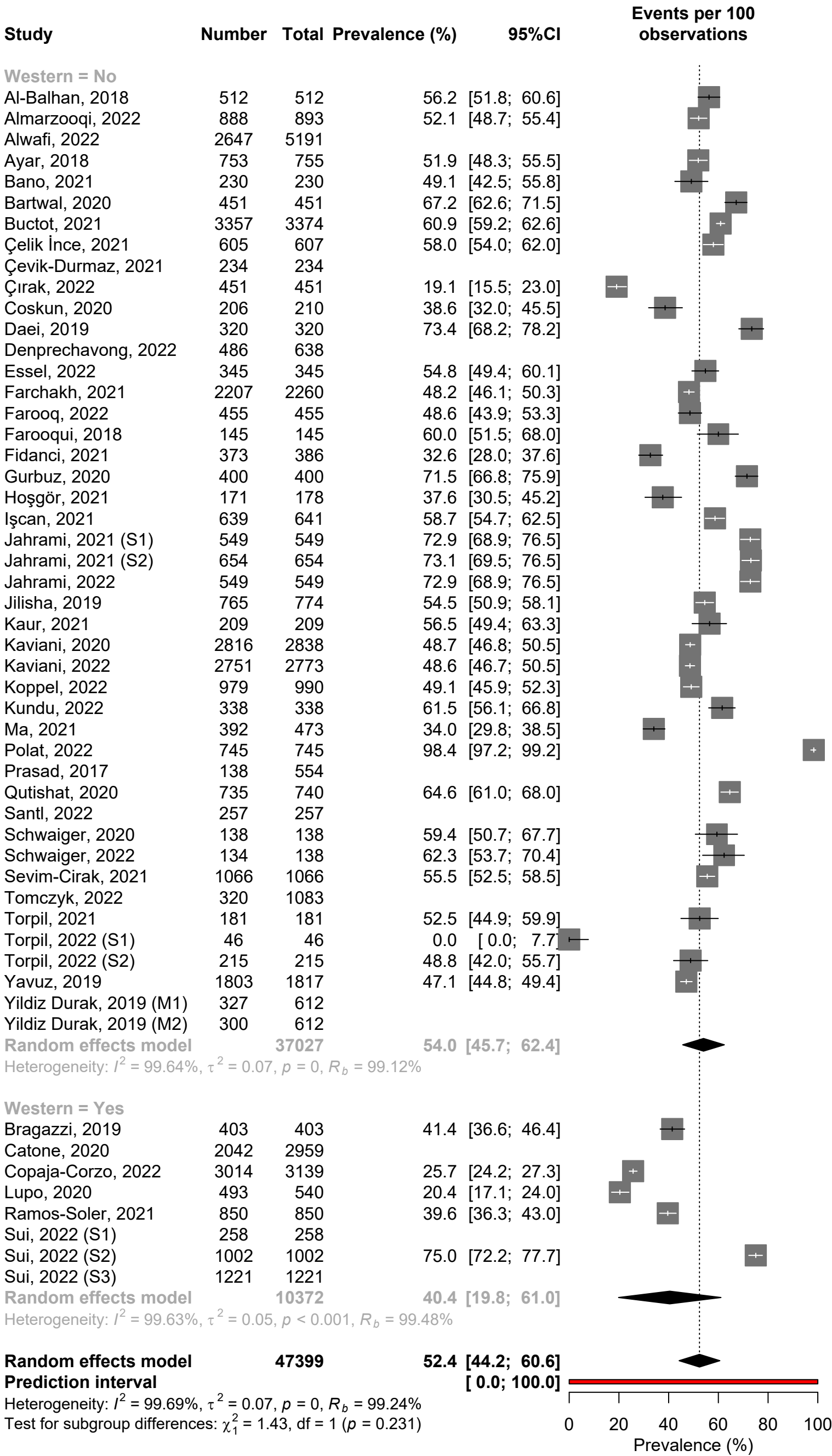

Supplement: Supplementary file 1 [file behavsci-13-00035-s001.zip › Supp S19.pdf]

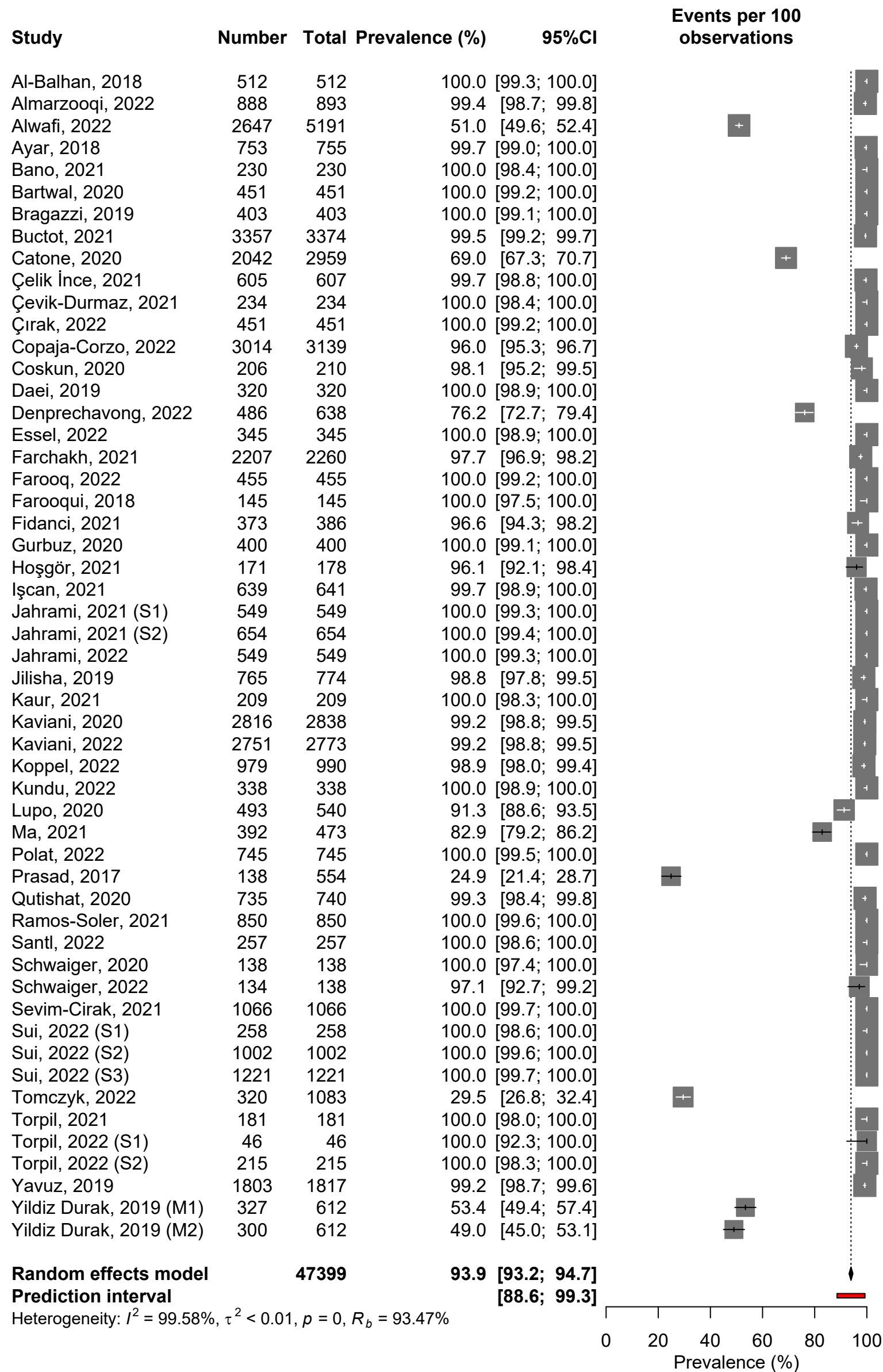

Supplement: Supplementary file 1 [file behavsci-13-00035-s001.zip › Supp S2.pdf]

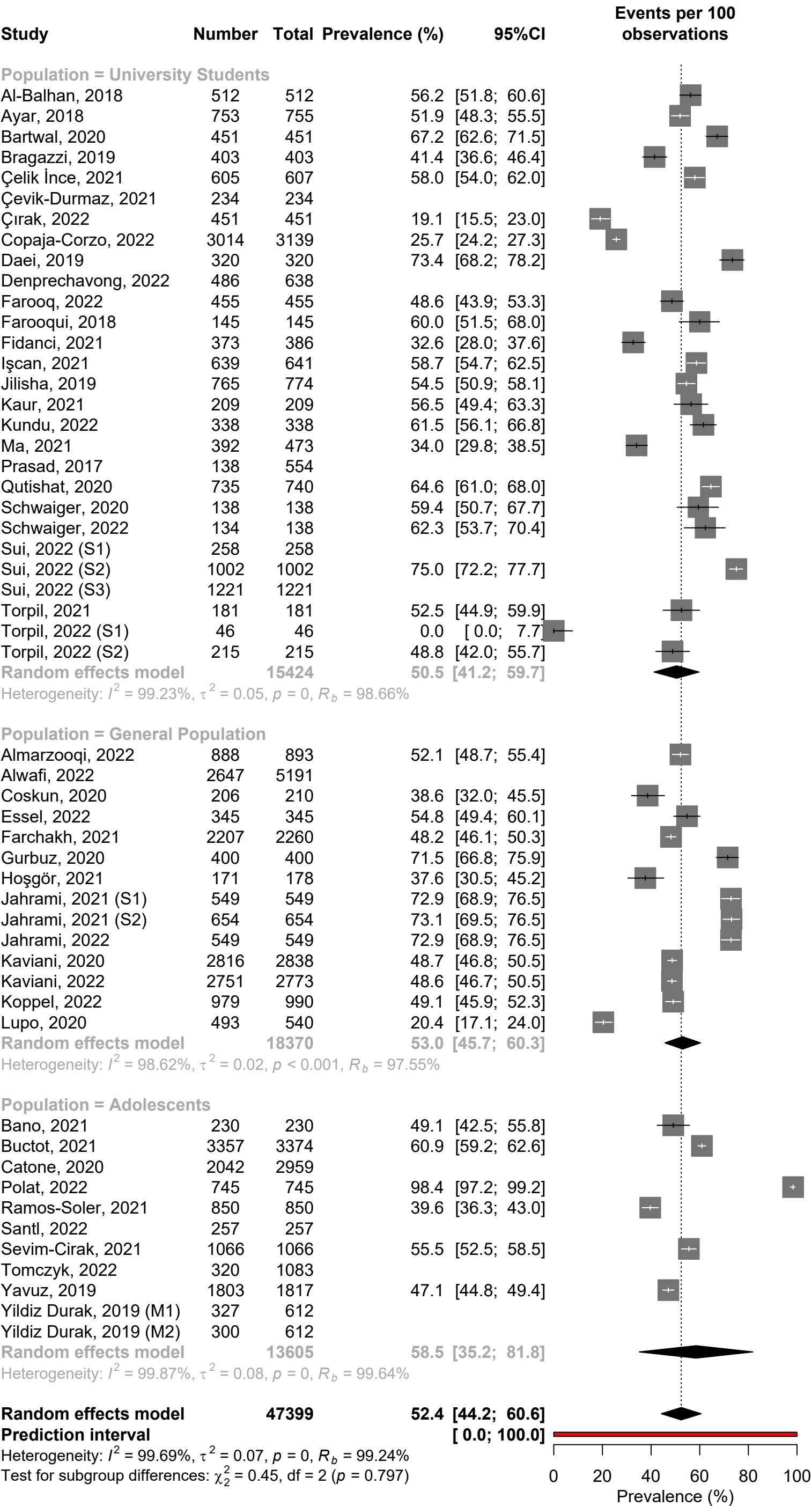

Supplement: Supplementary file 1 [file behavsci-13-00035-s001.zip › Supp S20.pdf]

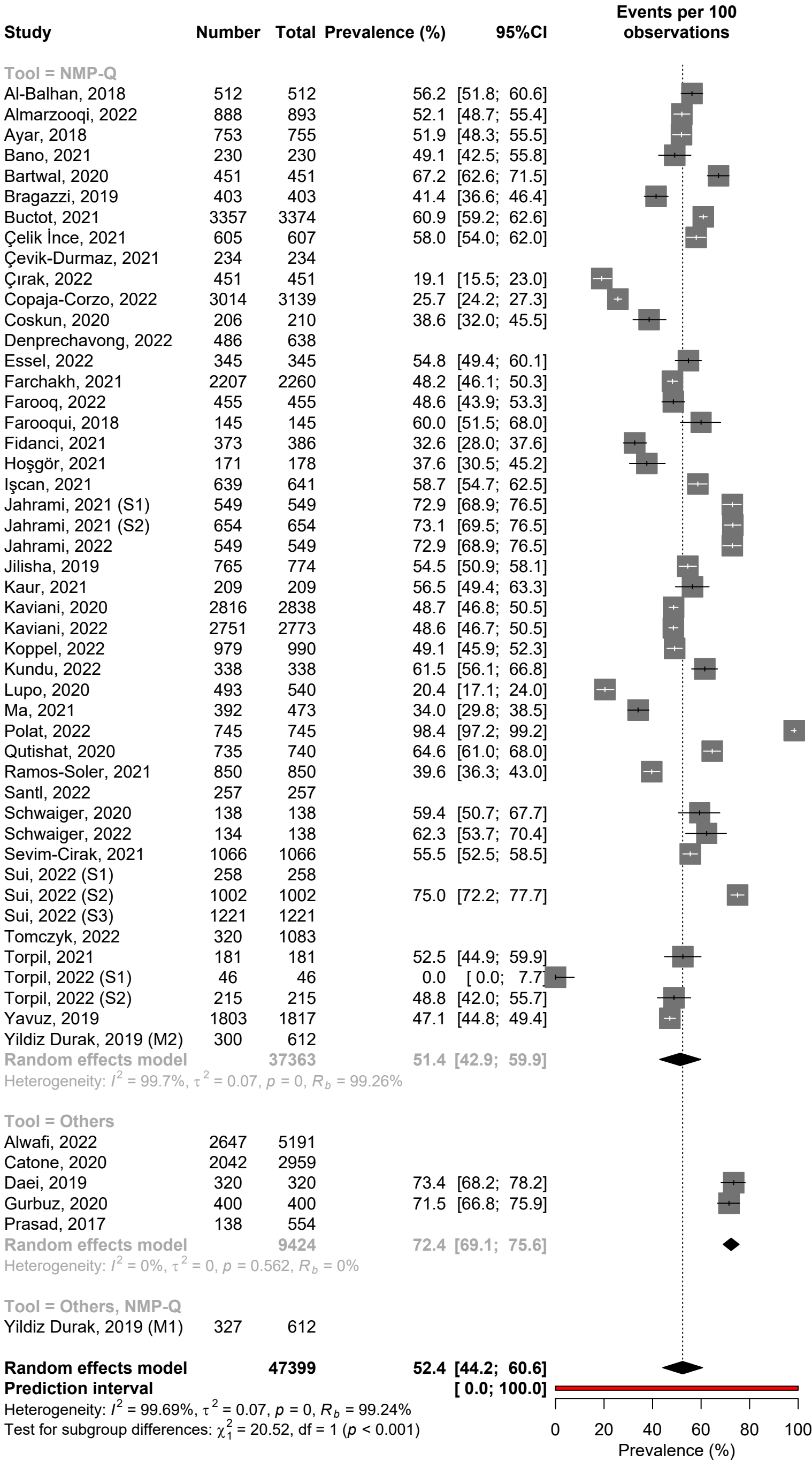

Supplement: Supplementary file 1 [file behavsci-13-00035-s001.zip › Supp S21.pdf]

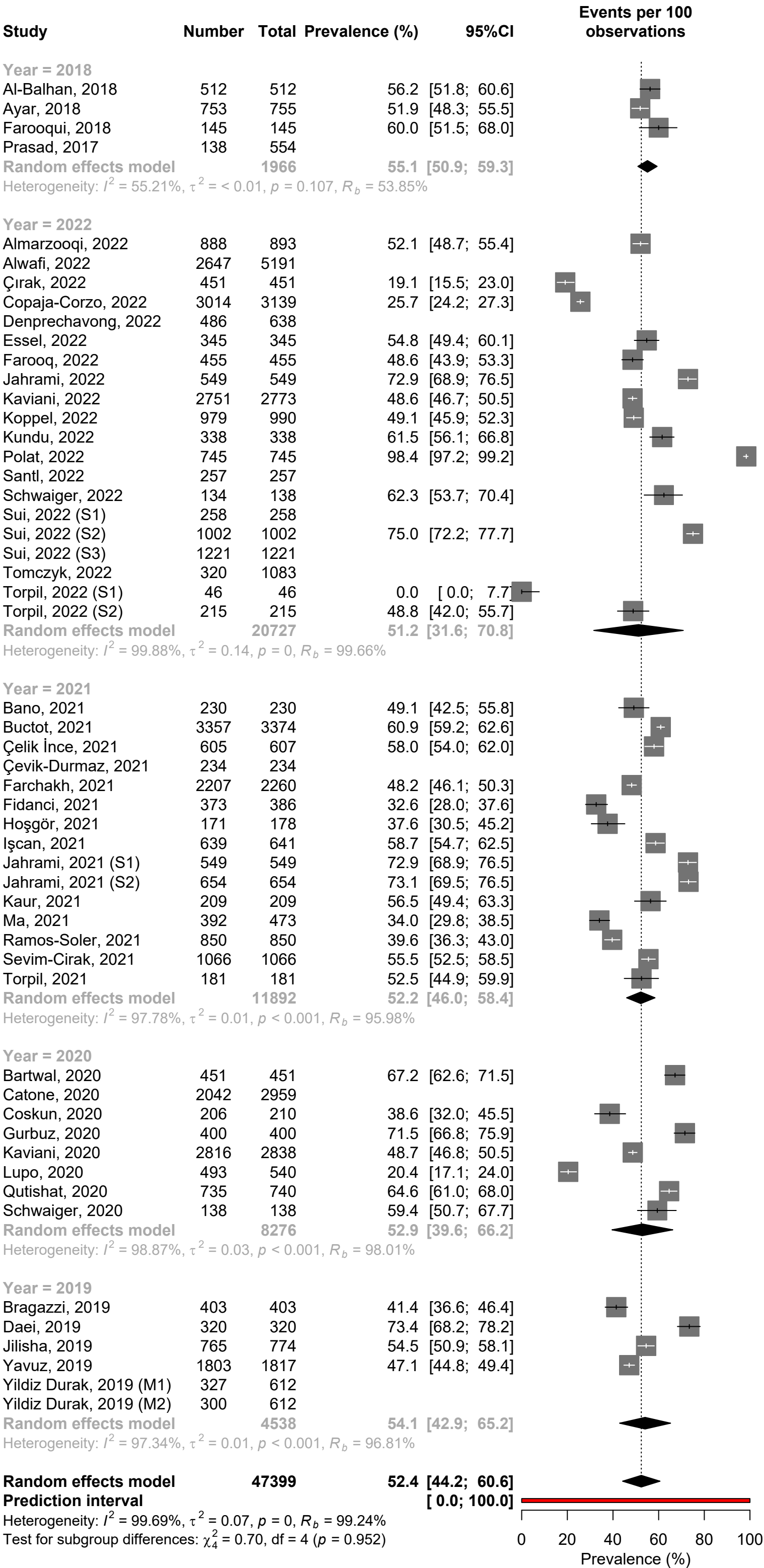

Supplement: Supplementary file 1 [file behavsci-13-00035-s001.zip › Supp S22.pdf]

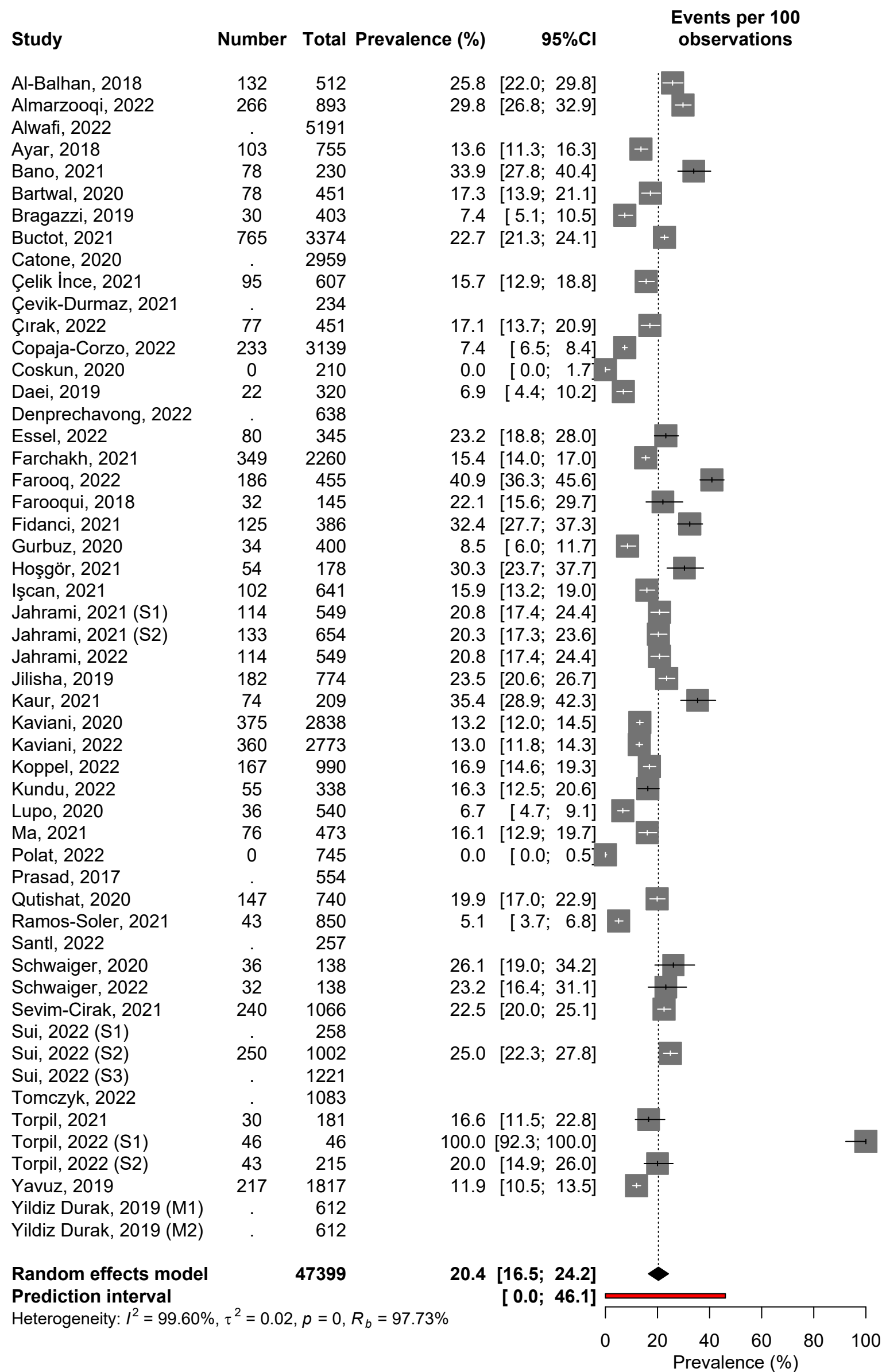

Supplement: Supplementary file 1 [file behavsci-13-00035-s001.zip › Supp S23.pdf]

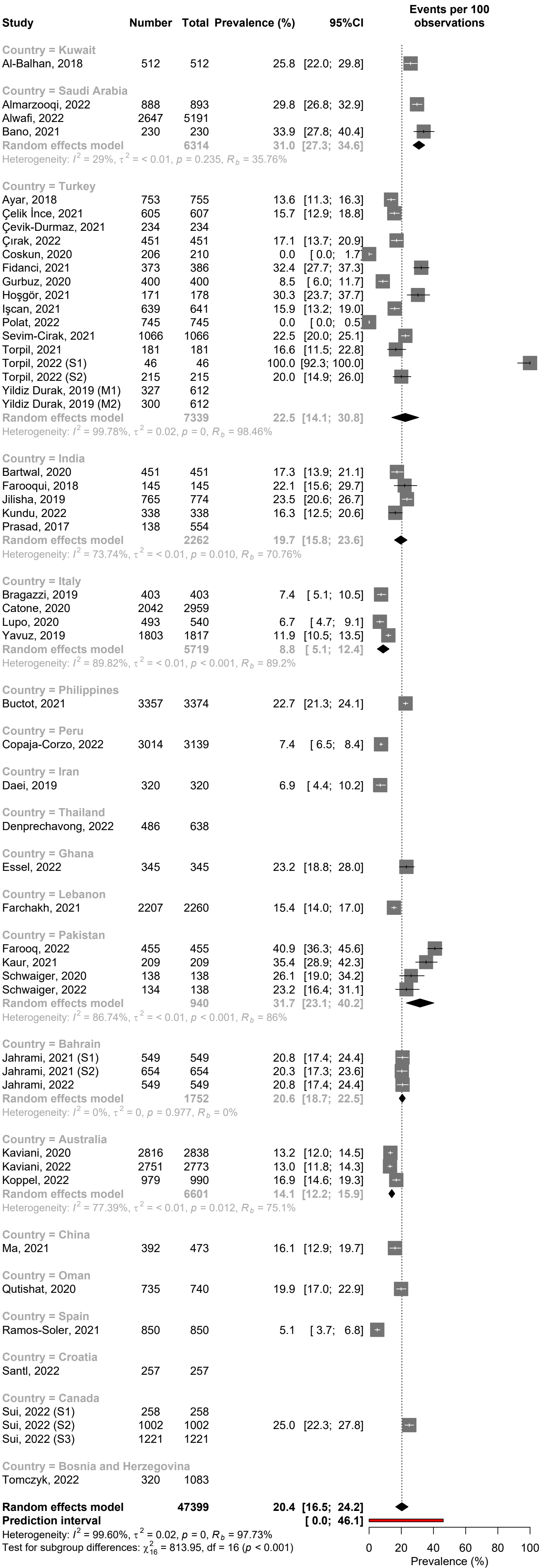

Supplement: Supplementary file 1 [file behavsci-13-00035-s001.zip › Supp S24.pdf]

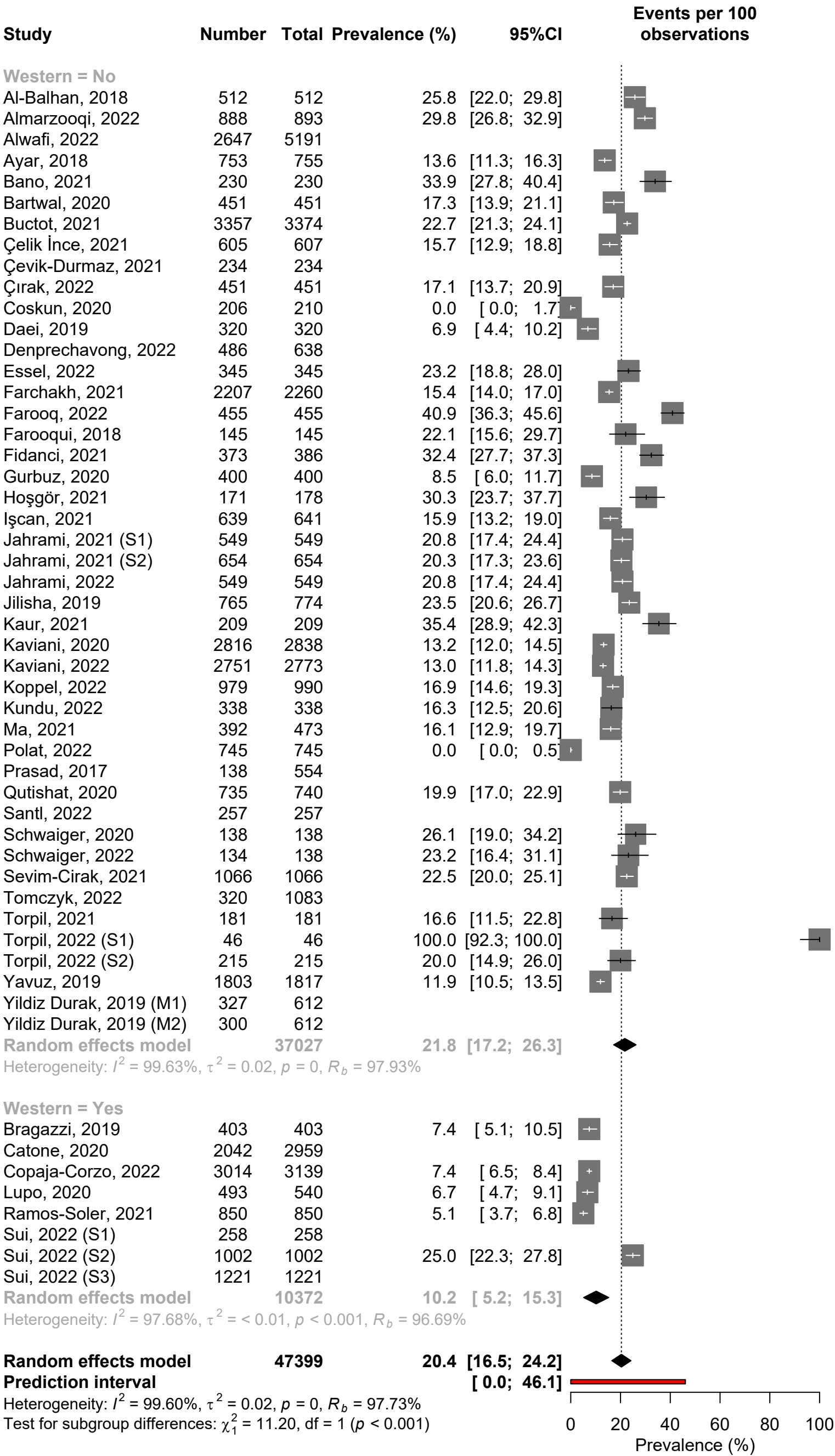

Supplement: Supplementary file 1 [file behavsci-13-00035-s001.zip › Supp S25.pdf]

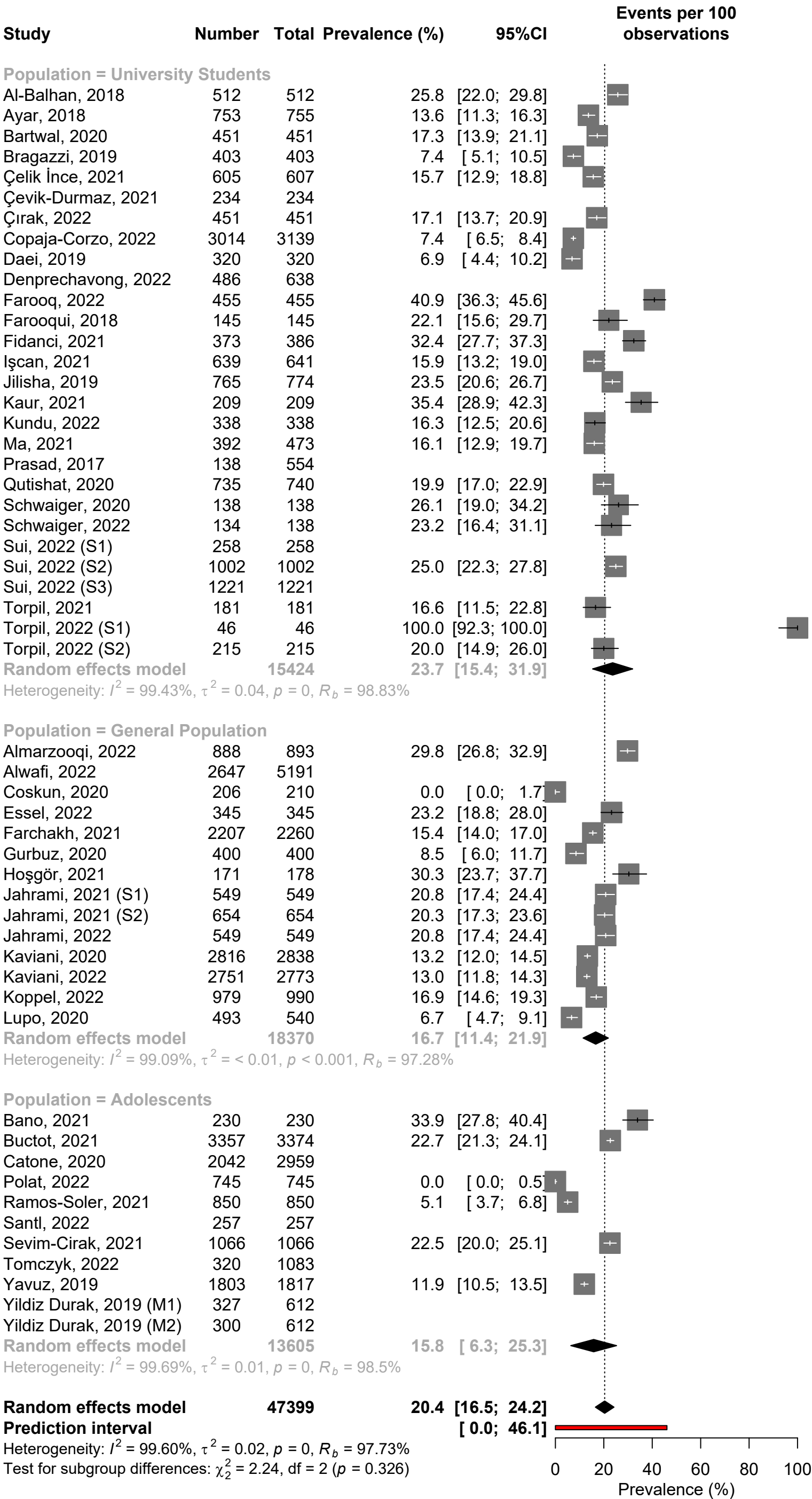

Supplement: Supplementary file 1 [file behavsci-13-00035-s001.zip › Supp S26.pdf]

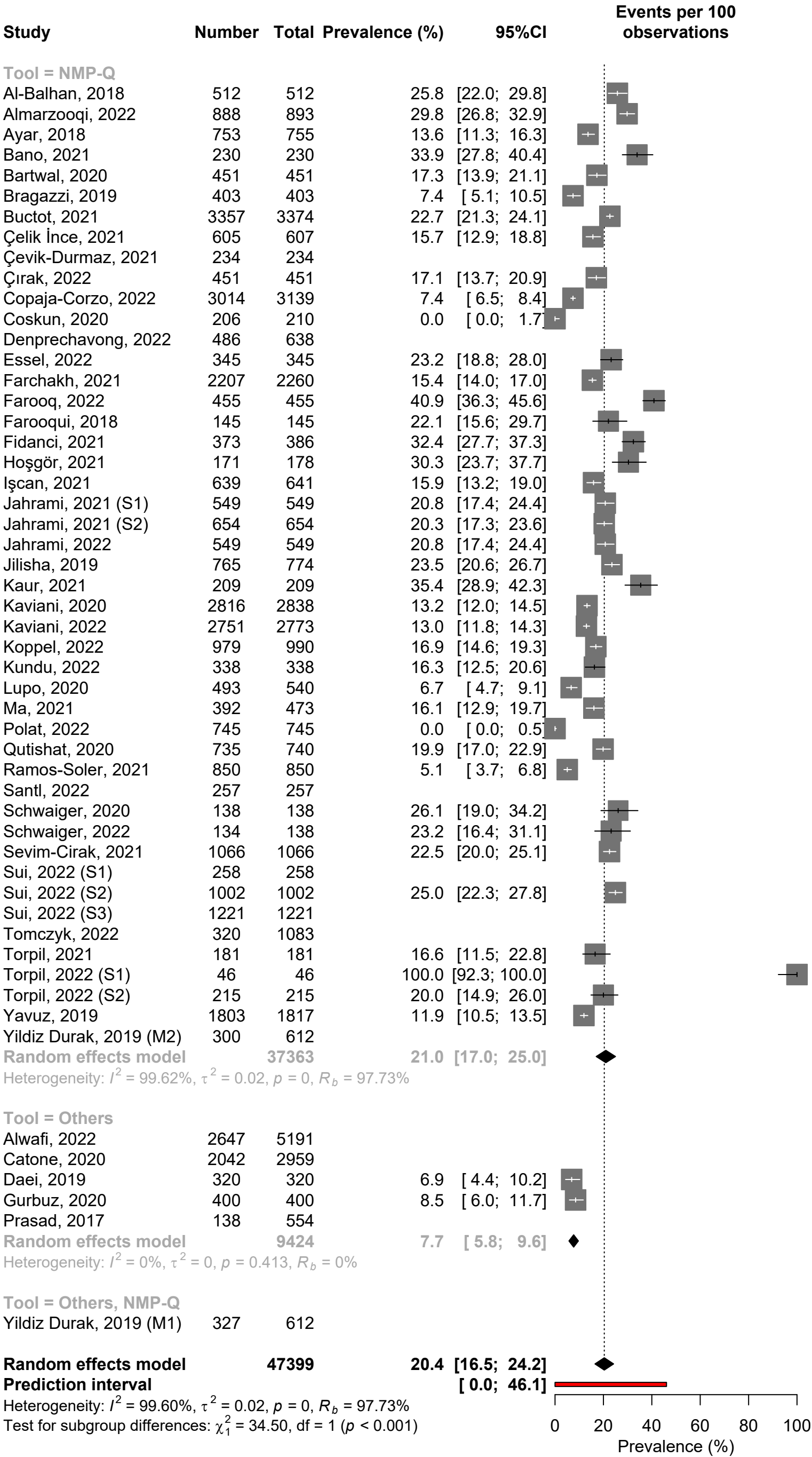

Supplement: Supplementary file 1 [file behavsci-13-00035-s001.zip › Supp S27.pdf]

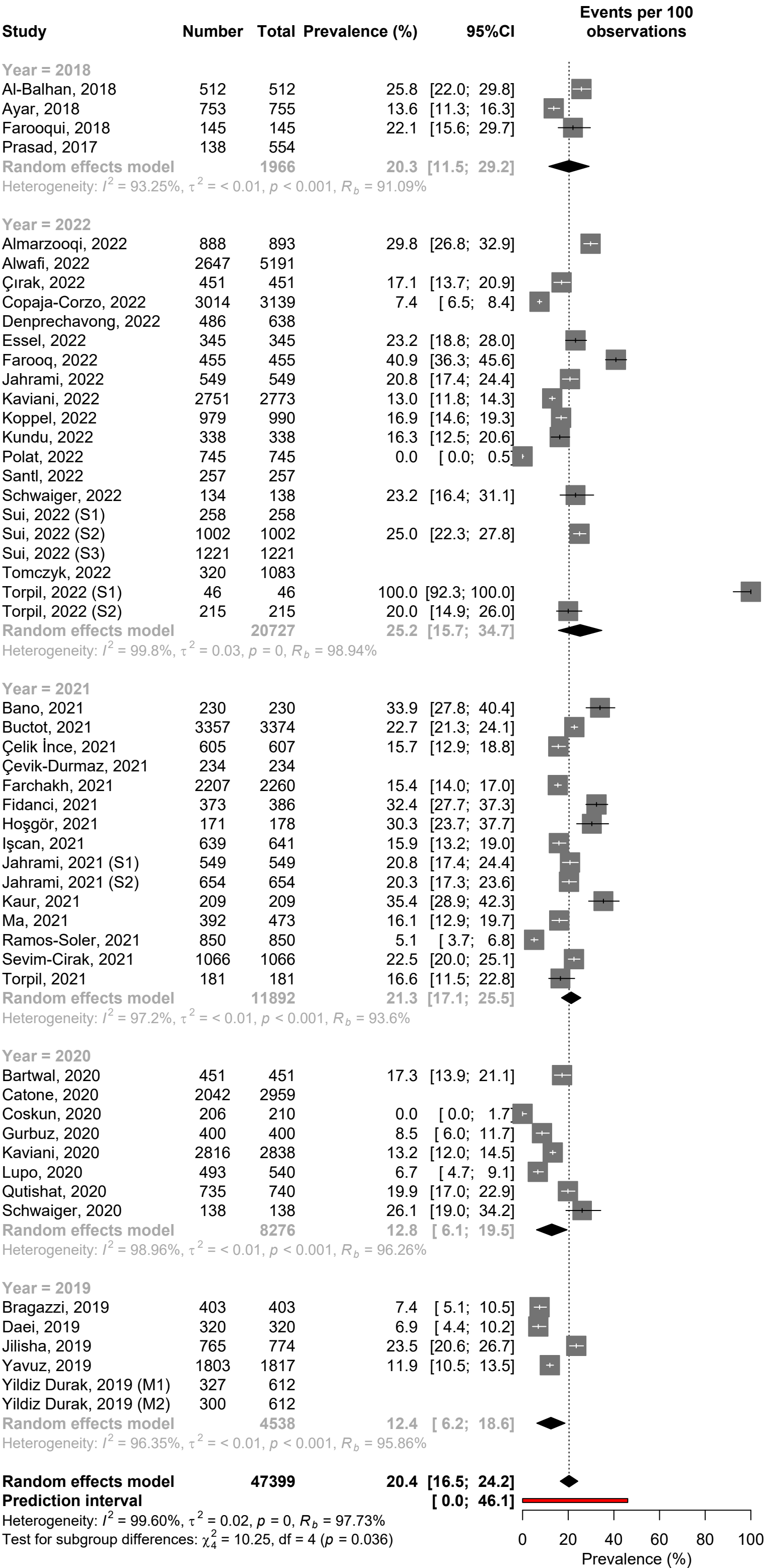

Supplement: Supplementary file 1 [file behavsci-13-00035-s001.zip › Supp S28.pdf]

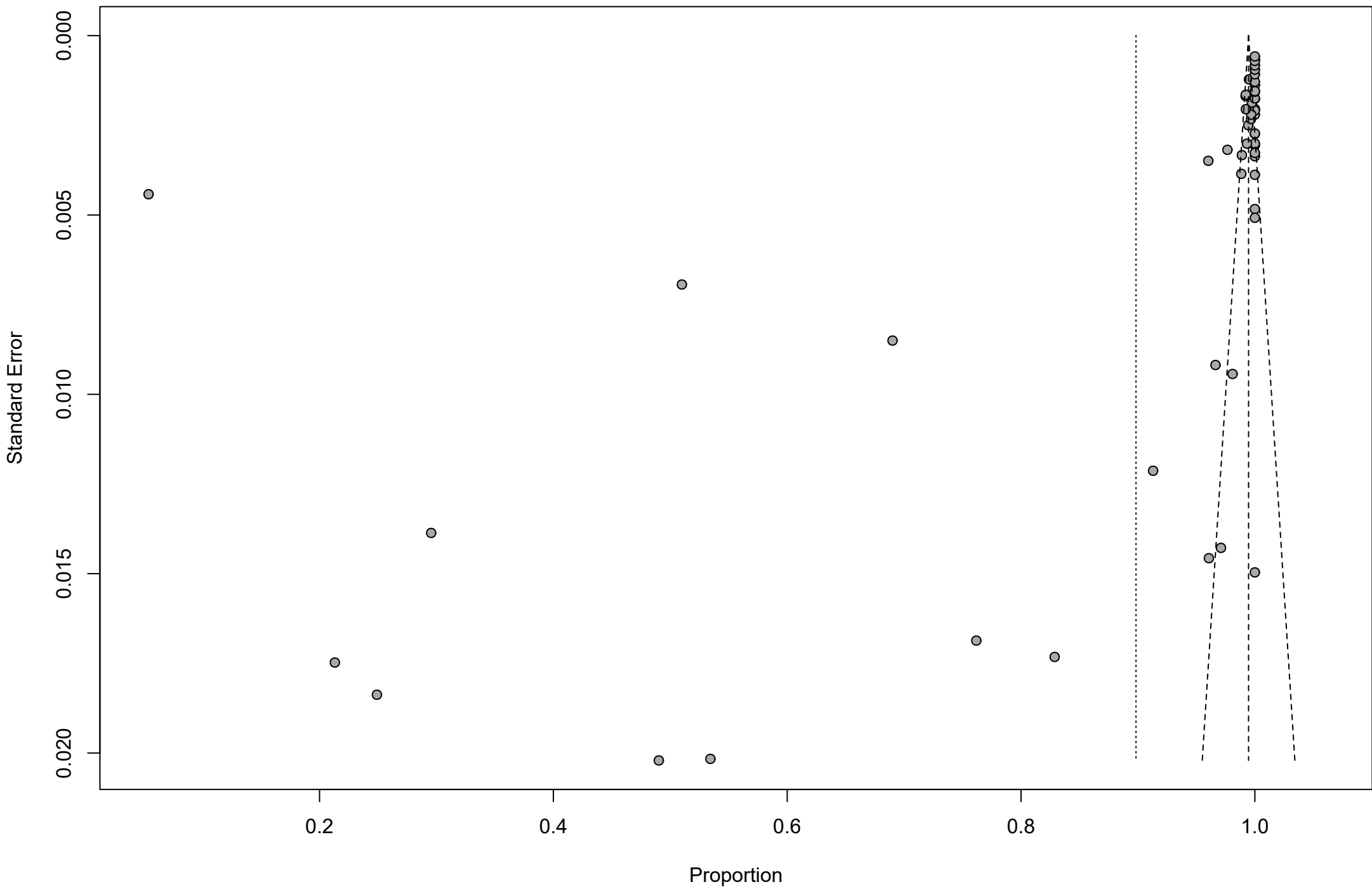

Supplement: Supplementary file 1 [file behavsci-13-00035-s001.zip › Supp S3.pdf]

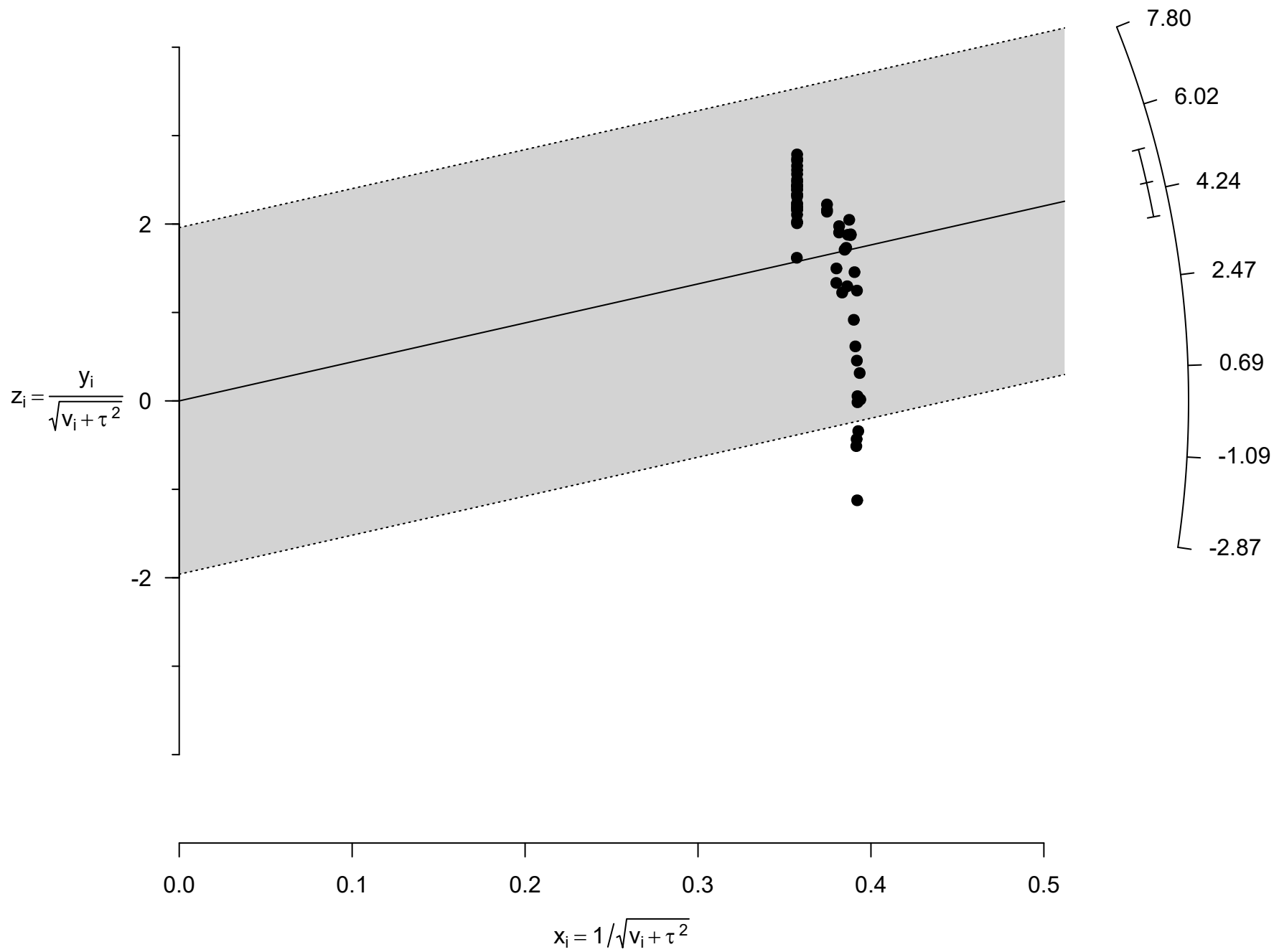

Supplement: Supplementary file 1 [file behavsci-13-00035-s001.zip › Supp S4.pdf]

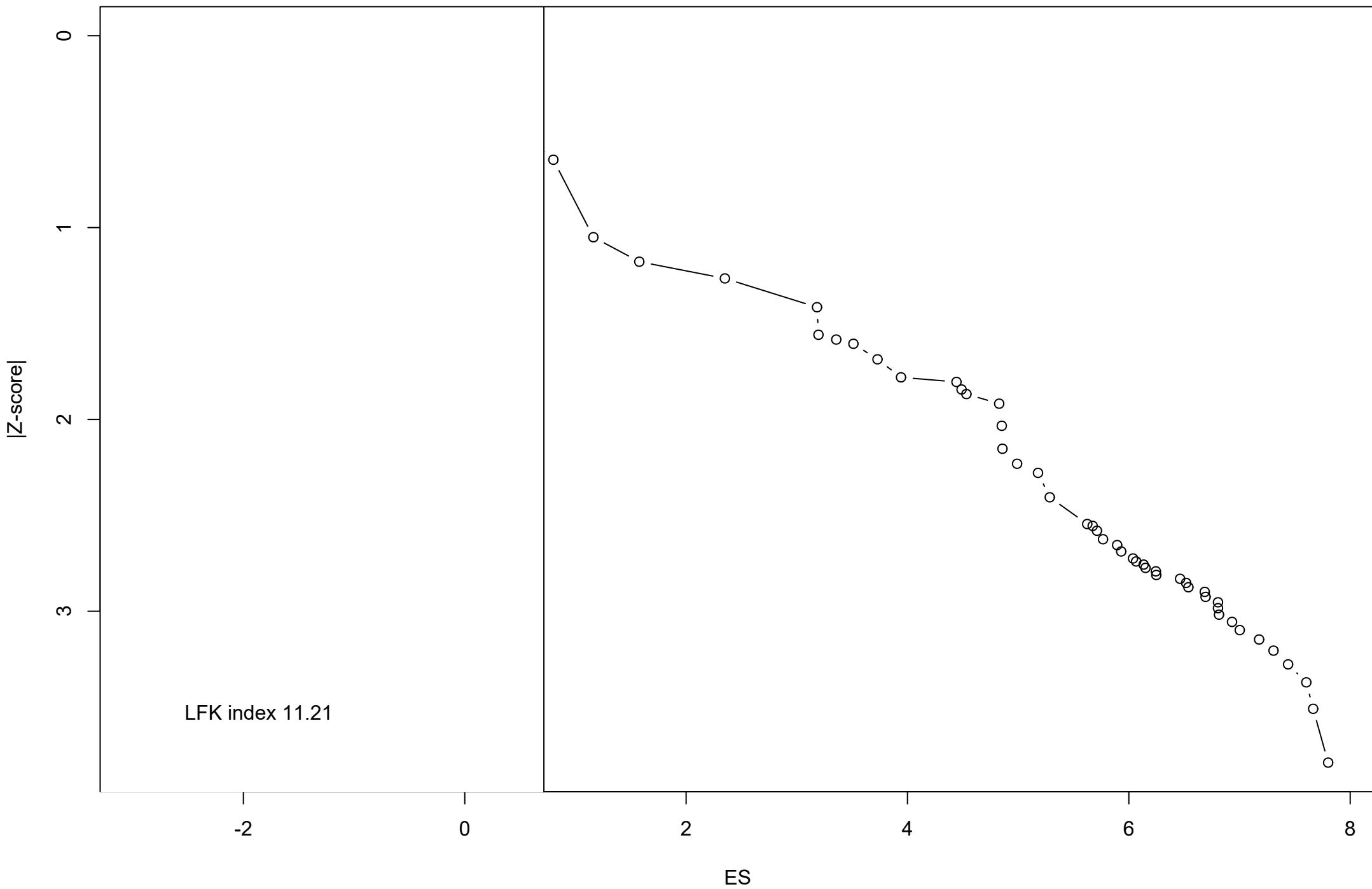

Supplement: Supplementary file 1 [file behavsci-13-00035-s001.zip › Supp S5.pdf]

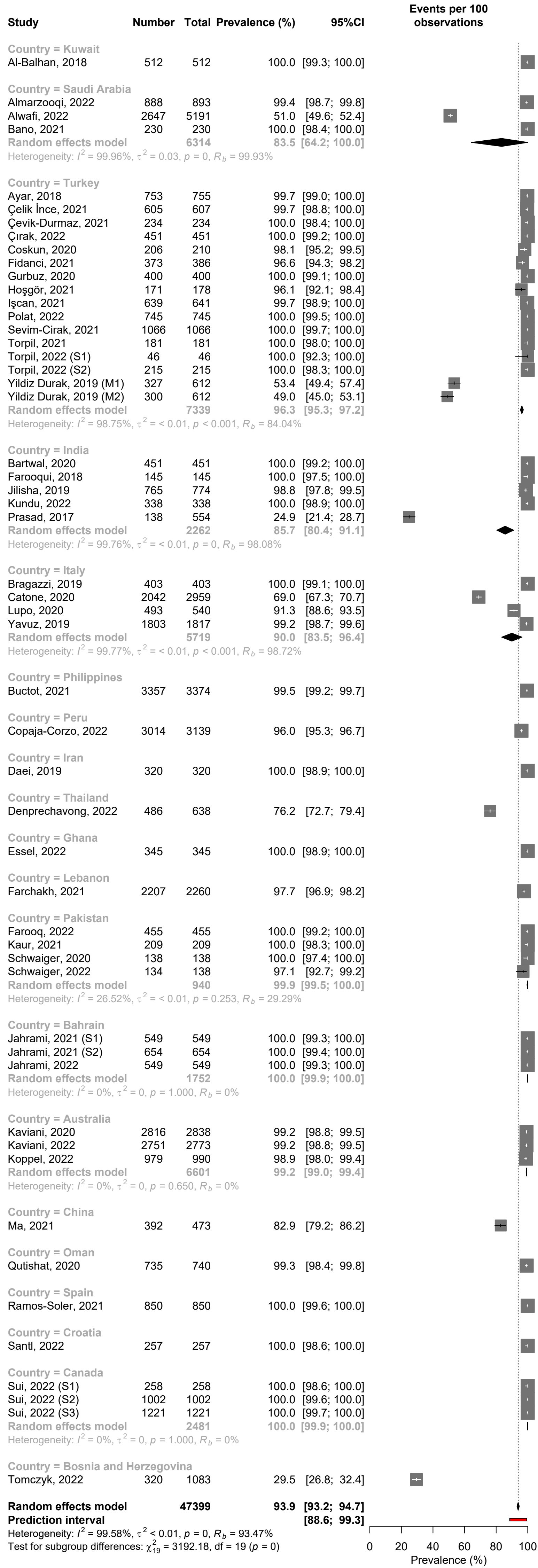

Supplement: Supplementary file 1 [file behavsci-13-00035-s001.zip › Supp S6.pdf]

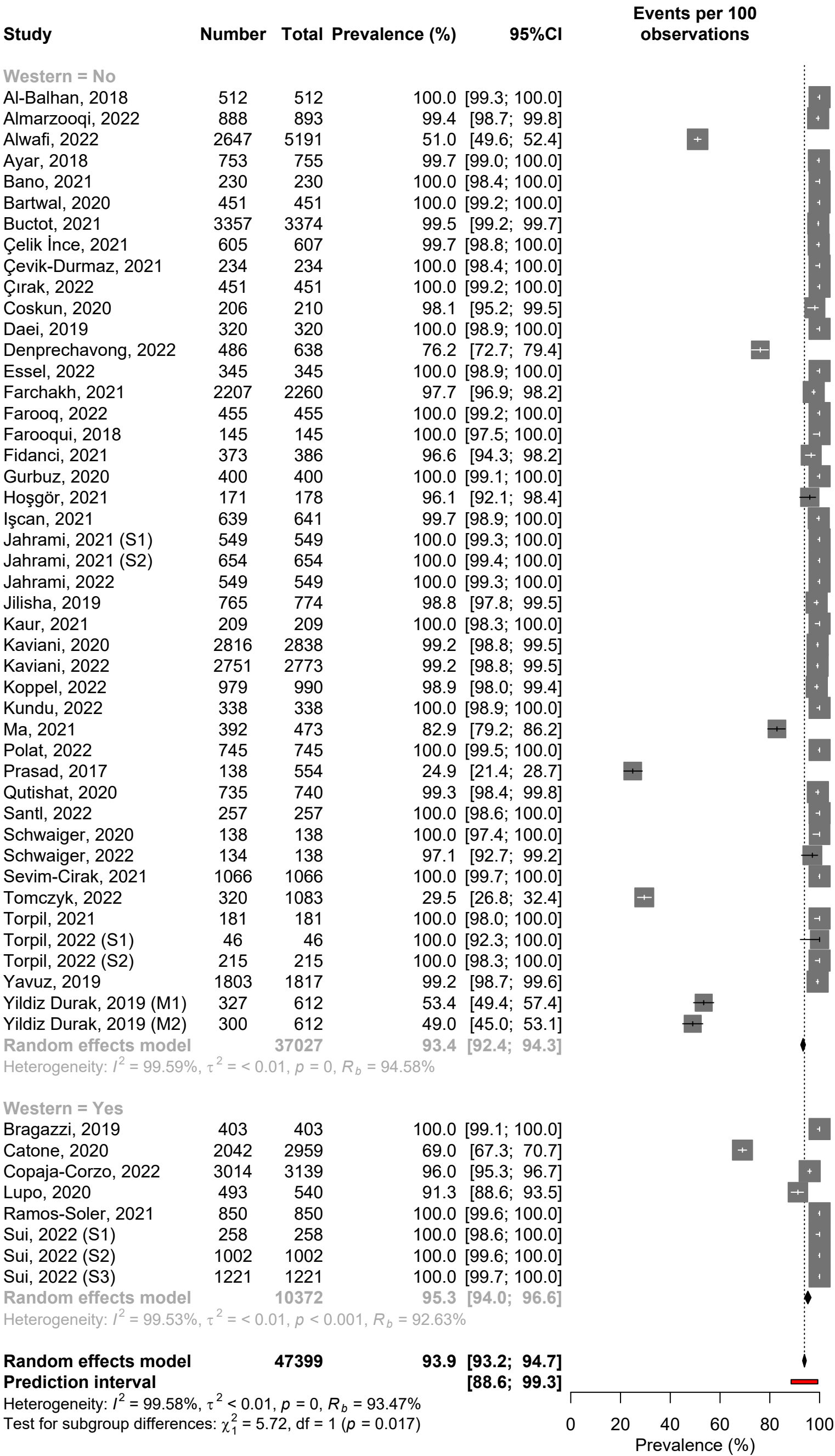

Supplement: Supplementary file 1 [file behavsci-13-00035-s001.zip › Supp S7.pdf]

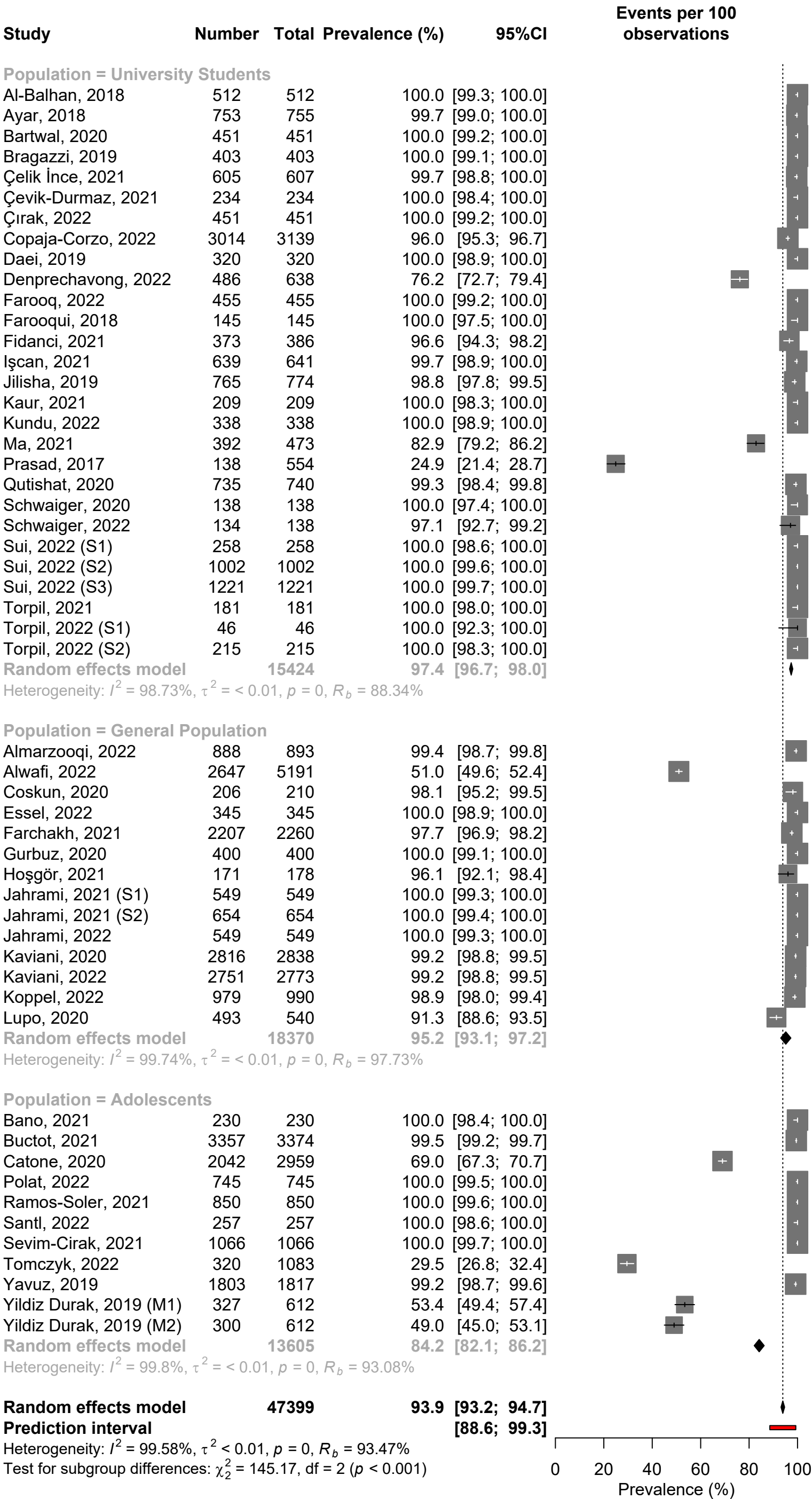

Supplement: Supplementary file 1 [file behavsci-13-00035-s001.zip › Supp S8.pdf]

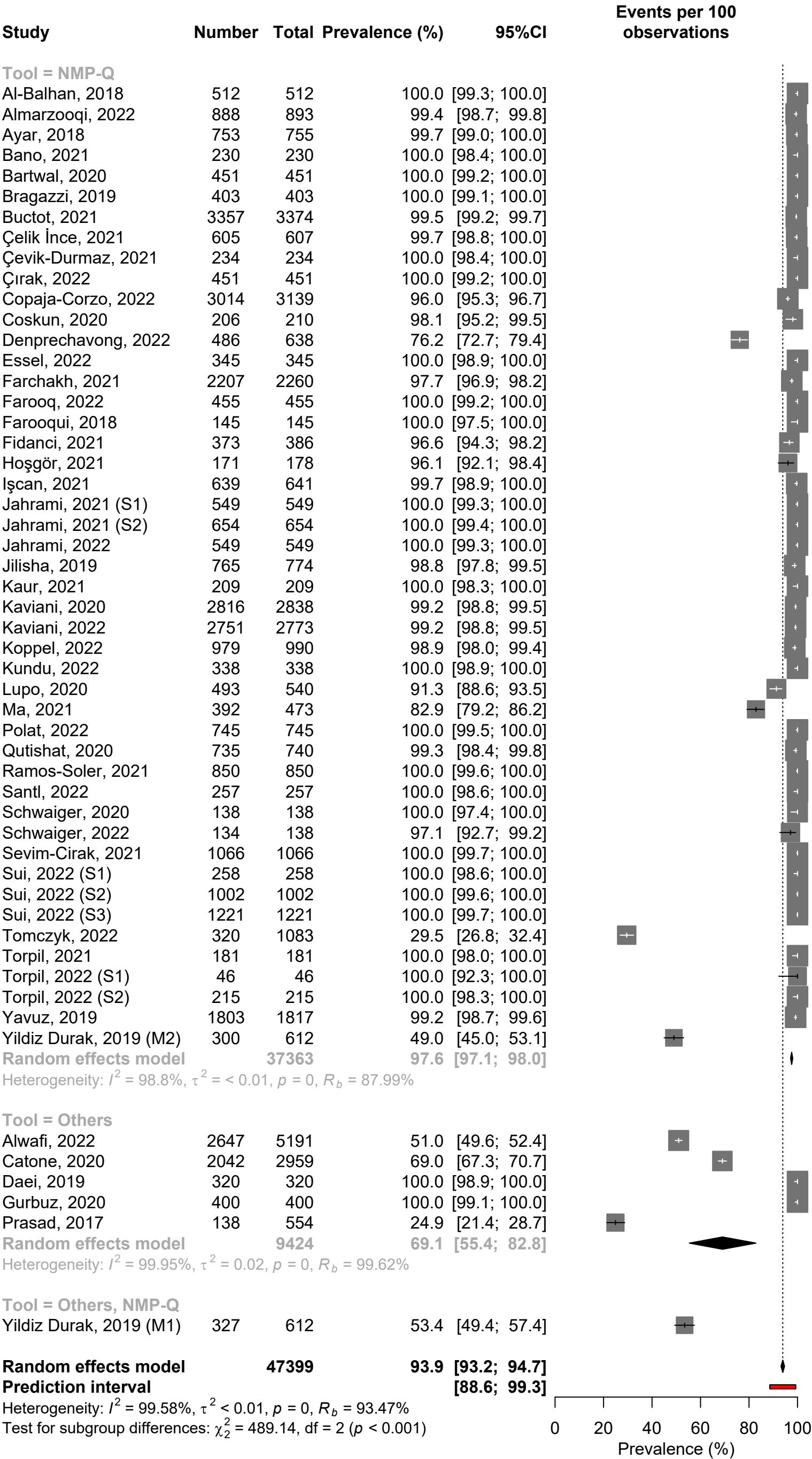

Supplement: Supplementary file 1 [file behavsci-13-00035-s001.zip › Supp S9.pdf]
